# Supplementary figures and images for: Analysis of the chemical constituents and their metabolites in Orthosiphon stamineus Benth. via UHPLC-Q exactive orbitrap-HRMS and AFADESI-MSI techniques
Source: PLoS One. 2024 Jun 25;19(6):e0304852. doi: 10.1371/journal.pone.0304852 (PMC11198764; doi:10.1371/journal.pone.0304852)

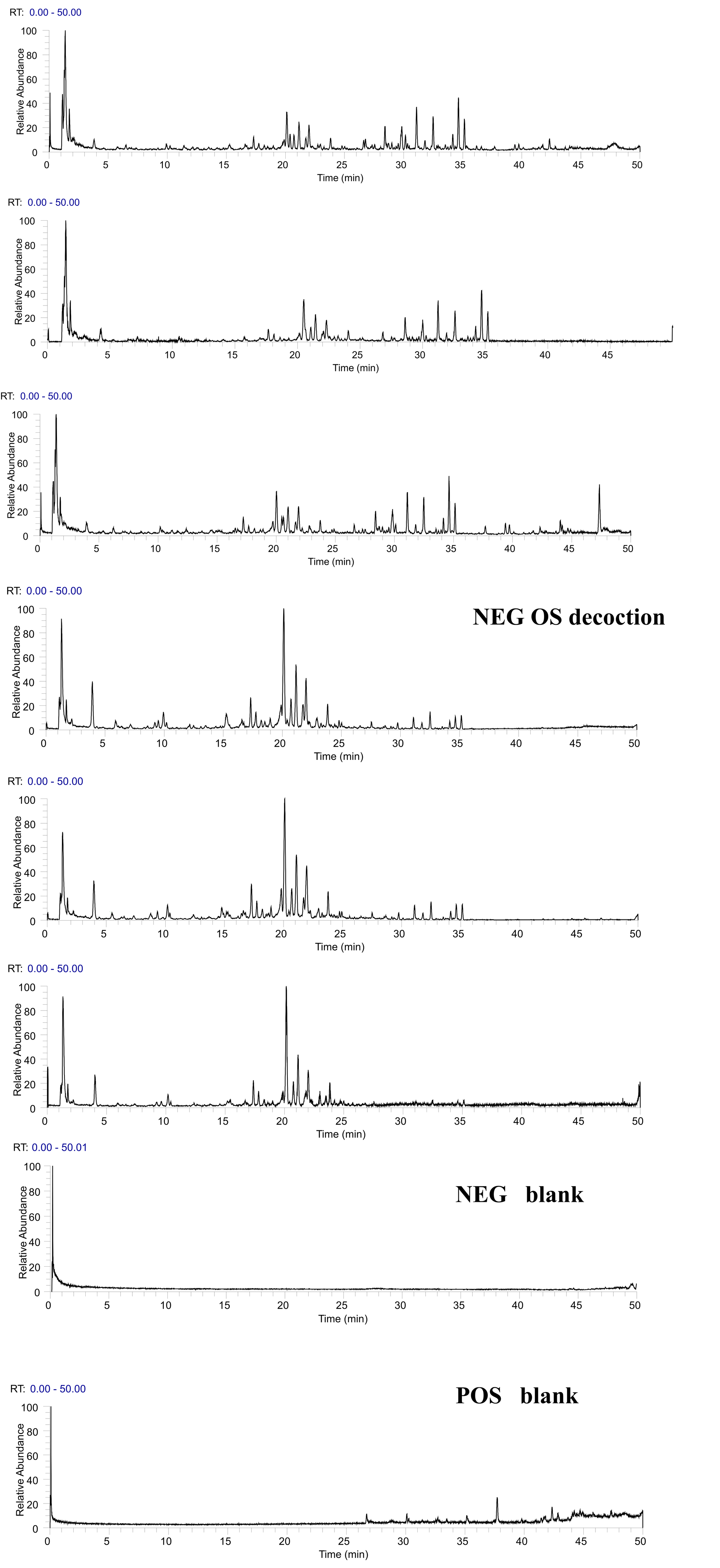

Supplement: S1 Fig — (TIF) [file pone.0304852.s001.tif]

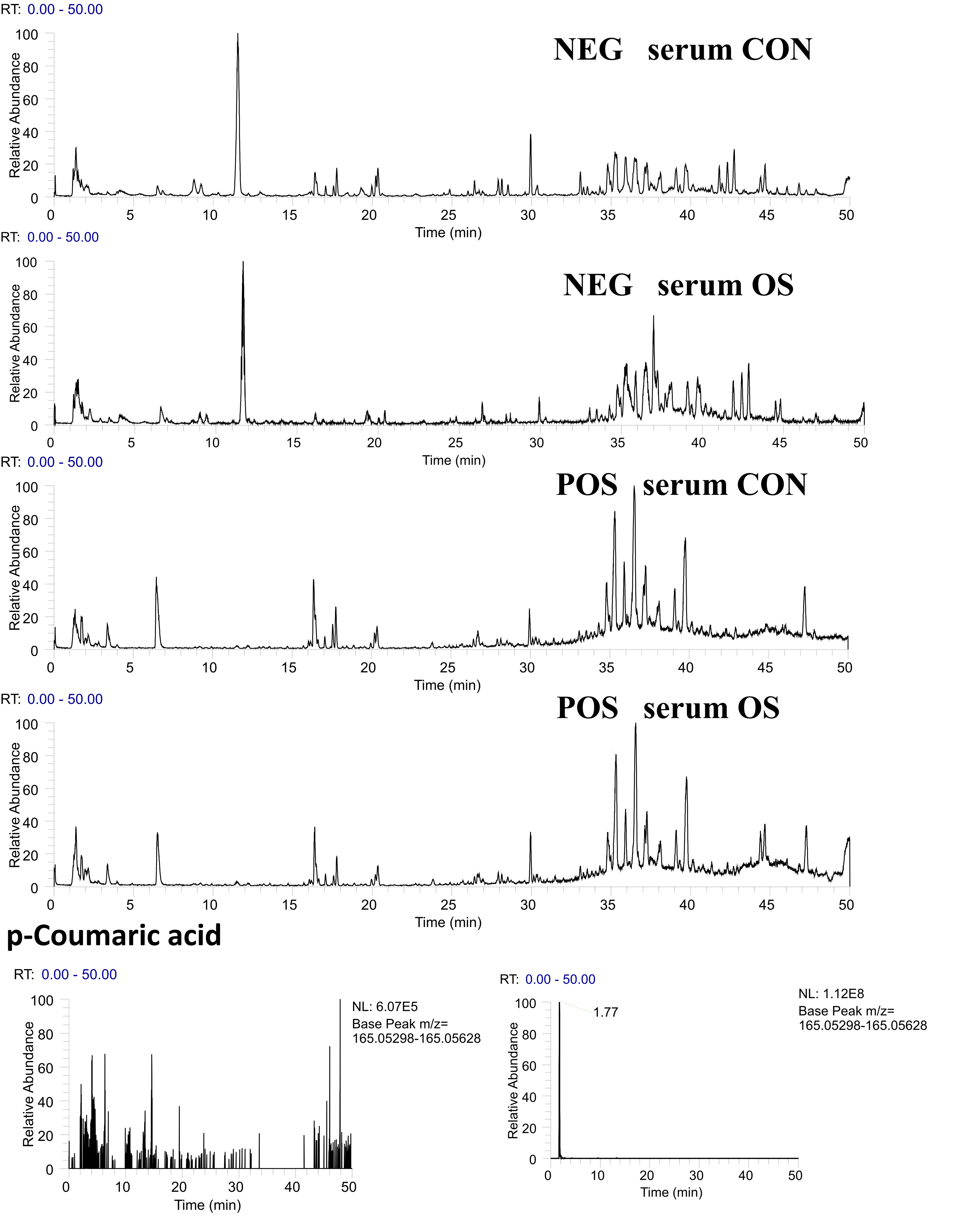

Supplement: S2 Fig — (TIF) [file pone.0304852.s002.tif]

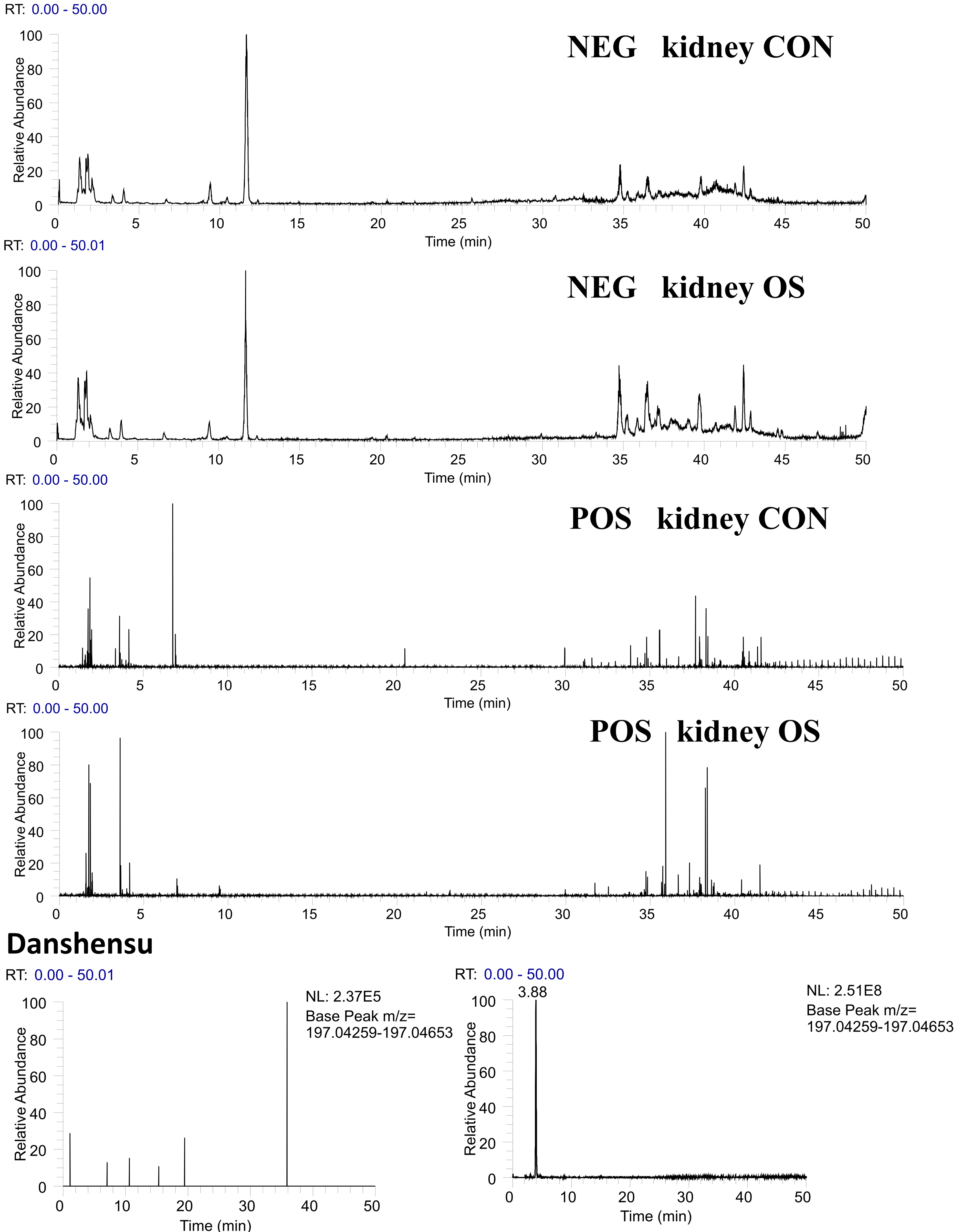

Supplement: S3 Fig — (TIF) [file pone.0304852.s003.tif]

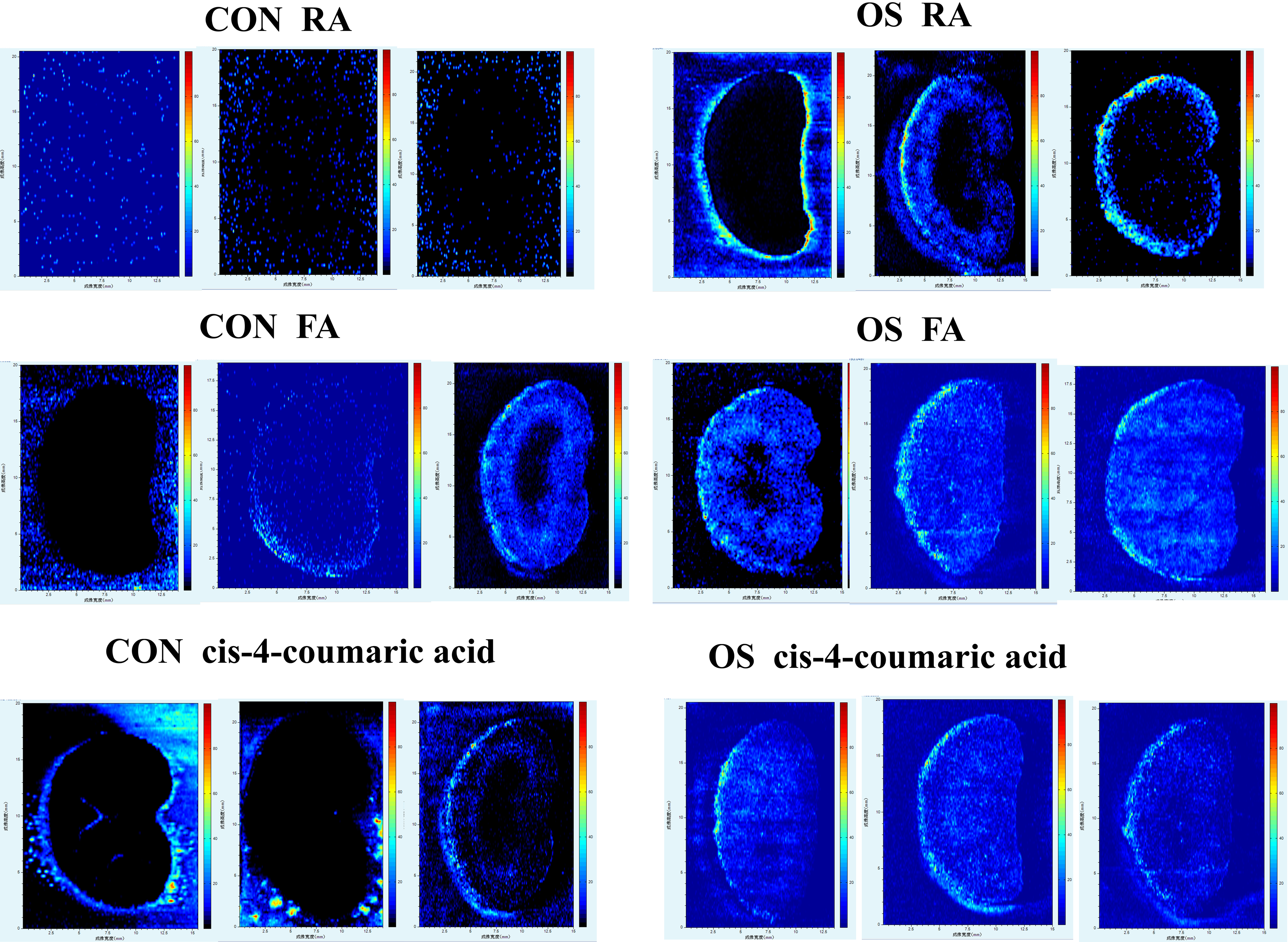

Supplement: S4 Fig — (TIF) [file pone.0304852.s004.tif]

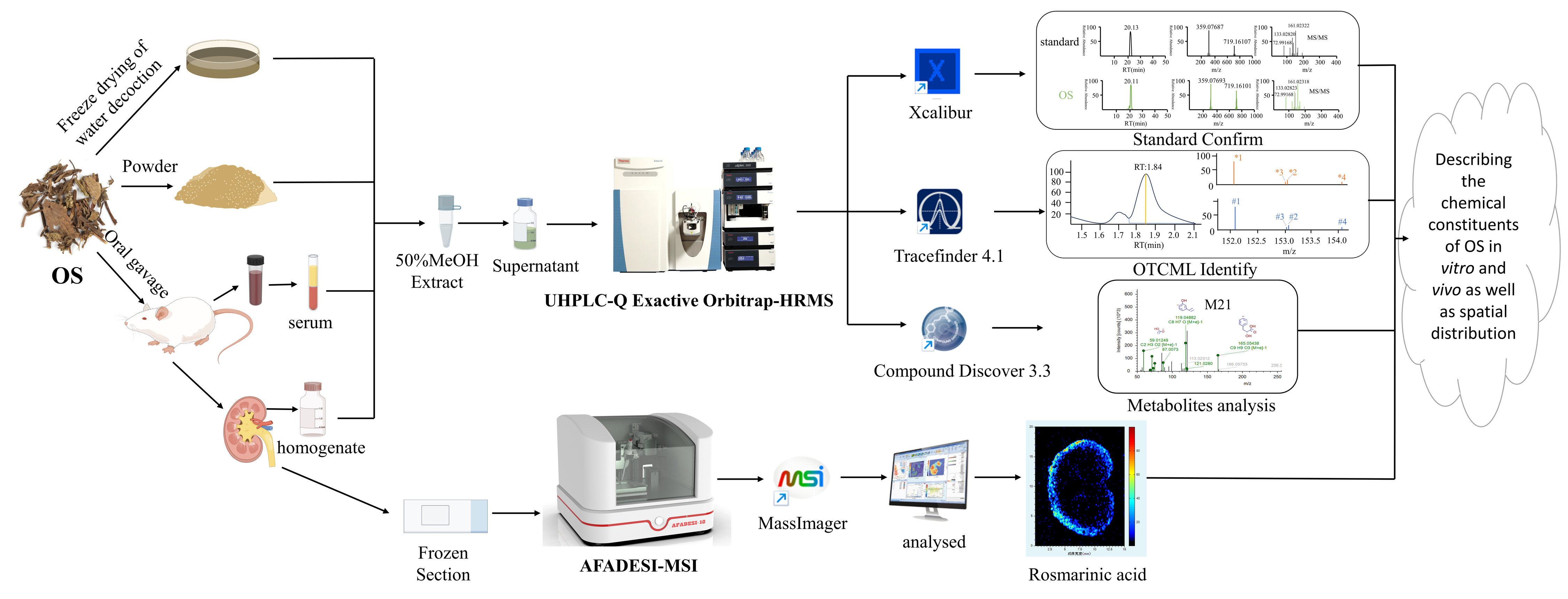

Supplement: S1 Graphical abstract — (TIF) [file pone.0304852.s008.tif]
