# Supplementary material for: Analysis of the chemical constituents and their metabolites in Orthosiphon stamineus Benth. via UHPLC-Q exactive orbitrap-HRMS and AFADESI-MSI techniques
Source: PLoS One. 2024 Jun 25;19(6):e0304852. doi: 10.1371/journal.pone.0304852 (PMC11198764; doi:10.1371/journal.pone.0304852)
Supplement: S1 Table — (DOCX) [file pone.0304852.s005.docx]

**S1 Table Reference Standards Purchase Information**

| **No.** | **reference standard** | **factory owners** | **location** | **lot numbers** |
| --- | --- | --- | --- | --- |
| 1 | Danshensu | Yuanye Bio-Technology | Shanghai, China | H09N10S102463 |
| 2 | protocatechuic acid | China National Insititutes for Drug Control | Beijing, China | 110809-202207 |
| 3 | Esculin | Vicky Biotechnology | Sichuan, China | wkq110729 |
| 4 | Protocatechualdehyde | Yuanye Bio-Technology | Shanghai, China | 110810-202210 |
| 5 | Chlorogenic acid | Must Bio-technology | Chengdu, Sichuan | MUST-170306201 |
| 6 | Vanillic acid | Must Bio-technology | Chengdu, Sichuan | MUST-22011113 |
| 7 | Cryptochlorogenic acid | Must Bio-technology | Chengdu, Sichuan | MUST-18032403 |
| 8 | Caffeic acid | China National Insititutes for Drug Control | Beijing, China | 110885-202007 |
| 9 | cis-4-coumaric acid | Must Bio-technology | Chengdu, Sichuan | MUST-18050603 |
| 10 | Sinapic acid | Yuanye Bio-Technology | Shanghai, China | SOSM6Y1 |
| 11 | ferulic acid | China National Insititutes for Drug Control | Beijing, China | 110773-201915 |
| 12 | Chicoric acid | Vicky Biotechnology | Sichuan, China | wkq21100909 |
| 13 | Rutin | Must Bio-technology | Chengdu, Sichuan | MUST-17122001 |
| 14 | Isoquercitrin | Psaitong Biotechnology | Beijing, China | C20PA98860C |
| 15 | Astragalin | Must Bio-technology | Chengdu, Sichuan | MUST-17031820 |
| 16 | Rosmarinic acid | China National Insititutes for Drug Control | Beijing, China | 111871-202007 |
| 17 | Lithospermic acid | Yuanye Bio-Technology | Shanghai, China | Y04J10H75487 |
| 18 | salvianolic acid B | Yuanye Bio-Technology | Shanghai, China | P13N11F130912 |
| 19 | Salvianolic acid A | Yuanye Bio-Technology | Shanghai, China | Z23D10X106625 |
| 20 | Salvianolic acid C | Must Bio-technology | Chengdu, Sichuan | MUST-18042102 |
| 21 | Sinensetin | Yuanye Bio-Technology | Shanghai, China | P04M11F109206 |
| 22 | Eupatorin | Yuanye Bio-Technology | Shanghai, China | Y31O10N101818 |
| 93 | 5-Hydroxymethylfurfural | Must Bio-technology | Chengdu, Sichuan | MUST-171215 |
| 94 | Neochlorogenic acid | Must Bio-technology | Chengdu, Sichuan | MUST ̄ 17011001 |
| 95 | (+)-Catechin Hydrate | Must Bio-technology | Chengdu, Sichuan | MUST-17060115 |
| 96 | Fraxin | Must Bio-technology | Chengdu, Sichuan | MUST-18051603 |
| 97 | puerarin | Yuanye Bio-Technology | Shanghai, China | MUST-1711007 |
| 98 | Tangshenoside I | Desite | Chengdu, China | DST-200815-190 |
| 99 | Scopoletin | Must Bio-technology | Chengdu, Sichuan | MUST-18052420 |
| 100 | Ellagic acid | Must Bio-technology | Chengdu, Sichuan | MUST-18052603 |
| 101 | Hyperoside | Vicky Biotechnology | Sichuan, China | wkq20031109 |
| 102 | Liquiritin apioside | Must Bio-technology | Chengdu, Sichuan | MUST-18123101 |
| 103 | Luteolin-7-O-glucoside | Must Bio-technology | Chengdu, Sichuan | MUST-21111817 |
| 104 | tectoridin | Must Bio-technology | Chengdu, Sichuan | MUST-22080204 |
| 105 | Narcissoside | Push Bio-technology | Chengdu, China | PS1887-0010MG |
| 106 | Quercitrin | Vicky Biotechnology | Sichuan, China | wkq20031704 |
| 107 | Naringin | Vicky Biotechnology | Sichuan, China | wkq111119 |
| 108 | Liquiritigenin | Extrasynthese Chemical | France | 30062-69727 |
| 109 | Lobetyolin | Must Bio-technology | Chengdu, Sichuan | MUST-22061005 |
| 110 | Isoliquiritin | Must Bio-technology | Chengdu, Sichuan | MUST-18031204 |
| 111 | Baicalin | Must Bio-technology | Chengdu, Sichuan | MUST-20030408 |
| 112 | Ononin | Vicky Biotechnology | Sichuan, China | wkq20060507 |
| 113 | Liquiritigenin | Must Bio-technology | Chengdu, Sichuan | MUST-18032104 |
| 114 | Luteolin | Must Bio-technology | Chengdu, Sichuan | MUST-22072315 |
| 115 | Quercetin | Must Bio-technology | Chengdu, Sichuan | MUST-22042012 |
| 116 | Calycosin | Must Bio-technology | Chengdu, Sichuan | MUST-21101117 |
| 117 | 3,8-Di-O-methylellagic acid | Desite | Chengdu, China | DST-180811-020 |
| 118 | Genistein | Must Bio-technology | Chengdu, Sichuan | MUST-20041422 |
| 119 | Naringenin | Must Bio-technology | Chengdu, Sichuan | MUST-18032406 |
| 120 | Apigenin | Must Bio-technology | Chengdu, Sichuan | MUST22030615 |
| 121 | Kaempferol | Must Bio-technology | Chengdu, Sichuan | MUST-22071311 |
| 122 | Isoliquiritigenin | Must Bio-technology | Chengdu, Sichuan | MUST-18011006 |
| 123 | Formononetin | Vicky Biotechnology | Sichuan, China | wkq20062208 |
| 124 | Medicarpin | Vicky Biotechnology | Sichuan, China | wkq20070203 |
| 125 | Chrysin | Aladdin Chemistry | Shanghai, China | 41219 |
| 126 | 6-Gingerol | MeilunBio | Dalian, China | S0609AS |
| 127 | oroxylin A | Nature Standard | Shanghai, China | 8387 |
| 128 | Licoricesaponin G2 | Vicky Biotechnology | Sichuan, China | wkq21052407 |
| 129 | Glycyrrhizic acid | Must Bio-technology | Chengdu, Sichuan | MUST-17060805 |
| 130 | Glycyrrhetinic acid | China National Insititutes for Drug Control | Beijing, China | 110723-200411 |
