# Supplementary material for: Analysis of the chemical constituents and their metabolites in Orthosiphon stamineus Benth. via UHPLC-Q exactive orbitrap-HRMS and AFADESI-MSI techniques
Source: PLoS One. 2024 Jun 25;19(6):e0304852. doi: 10.1371/journal.pone.0304852 (PMC11198764; doi:10.1371/journal.pone.0304852)
Supplement: S2 Table — (DOCX) [file pone.0304852.s006.docx]

**S2 Table Compounds confirmed as absent are tabulated**

| **No.** | **t/min** | **Plausible identity** | **Molecular formula** | **Neutral mass (Da)** | **Pseudo molecular ion** | **MS1(m/z)** | **MS2(m/z)** | **position** |
| --- | --- | --- | --- | --- | --- | --- | --- | --- |
| 93 | 3.52 | 5-Hydroxymethylfurfural | C_6_H_6_O_3_ | 126.11 | [M+H]+ | 127.039 | 53.03897，81.03349，54.04230，51.02333 | 3 |
|  | 3.64 |  |  |  | [M-H]- | 125.023 | 67.01740，79.01746，123.94485，95.01225 |  |
| 94 | 5.78 | Neochlorogenic acid | C_16_H_18_O_9_ | 354.309 | [M+H]+ | 355.1022 | 163.03891，135.04404，117.03352，89.03842 | 1 |
|  | 5.76 |  |  |  | [M-H]- | 353.0875 | 135.04385，191.05507，179.03384，85.02802 |  |
| 95 | 9.09 | (+)-Catechin Hydrate | C_15_H_16_O_7_ | 308.28 | [M+H-H2O]+ | 291.0853 | 289.06998,171.99254,139.03864 | 3 |
|  | 9.12 |  |  |  | [M-H-H20]- | 289.0713 | 123.04366，109.02805，126.02670，97.02797 |  |
| 96 | 10.68 | Fraxin | C_16_H_18_O_10_ | 370.308 | [M+H]+ | 371.097 | 209.0447 | 1 |
|  | 10.74 |  |  |  | [M-H]- | 369.0824 | 192.00534，207.02896，163.00261，135.0074 |  |
| 97 | 11.85 | puerarin | C_21_H_20_O_9_ | 416.4 | [M+H]+ | 417.1171 | 297.07523，267.06473，307.09534，281.09534 | 3 |
|  | 11.88 |  |  |  | [M-H]- | 415.1031 | 267.06601，295.06085，277.04990 |  |
| 98 | 14.54 | Tangshenoside I | C_29_H_42_O_18_ | 678.633 | [M+NH4]+ | 696.27 | 193.08582，161.05959，133.06468，105.06980 | 3 |
|  | 14.55 |  |  |  | [M-H]- | 677.2295 | 99.04376，261.09775，161.04437，100.04713 |  |
| 99 | 14.7 | Scopoletin | C_10_H_8_O_4_ | 192.168 | [M+H]+ | 193.0495 | 66.04662，94.04137，122.03634，133.02847 | 1 |
|  | 14.74 |  |  |  | [M-H]- | 191.034 | 176.01022，104.02536，148.01523，120.02015 |  |
| 100 | 15.41 | Ellagic acid | C_14_H_6_O_8_ | 302.193 | [M+H]+ | 303.0135 | 201.01833，257.00793，275.01855，229.01295 | 1 |
|  | 15.42 |  |  |  | [M-H]- | 300.9987 | 283.99588，229.01341，201.01833，145.02811 |  |
| 101 | 16.21 | Hyperoside | C_21_H_20_O_12_ | 464.376 | [M+H]+ | 465.1028 | 303.04950，85.02837，97.02835，69.03359 | 2 |
|  | 16.23 |  |  |  | [M-H]- | 463.088 | 300.02713，271.02444，255.02917，151.00244 |  |
| 102 | 16.55 | Liquiritin apioside | C_26_H_30_O_13_ | 550.5 | [M+H]+ | 551.1749 | 257.08029，137.02313，85.02834，147.04373 | 3 |
|  | 16.59 |  |  |  | [M-H]- | 549.1614 | 255.06592，135.00746，119.04878，153.01813 |  |
| 103 | 16.86 | Luteolin-7-O-glucoside | C_21_H_20_O_11_ | 448.377 | [M+H]+ | 449.1078 | 287.05490，153.01820，288.05823 | 1 |
|  | 16.96 |  |  |  | [M-H]- | 447.0928 | 285.03973，284.03223，286.04361 |  |
| 104 | 17.6 | tectoridin | C_22_H_22_O_11_ | 462.404 | [M+H]+ | 463.1303 | 301.07028，286.04681 | 2 |
|  | 17.64 |  |  |  | [M-H]- | 461.1085 | 283.02451，255.02928，299.05542，284.03094 |  |
| 105 | 18.56 | Narcissoside | C_28_H_32_O_16_ | 624.5 | [M+H]+ | 625.1752 | 317.06497，318.0683，85.02831，71.04913 | 3 |
|  | 18.58 |  |  |  | [M-H]- | 623.1613 | 315.05048，300.02618，271.02359，243.02840 |  |
| 106 | 18.64 | Quercitrin | C_21_H_20_O_11_ | 448.377 | [M+H]+ | 449.1075 | 303.04947，85.02834，71.04917，304.05267 | 2 |
|  | 18.68 |  |  |  | [M-H]- | 447.093 | 300.02710，271.02454，255.02927，151.00247 |  |
| 107 | 19.41 | Naringin | C_27_H_32_O_14_ | 580.5 | [M+H]+ | 581.1854 | 273.07529，85.02832，71.04914，153.01805 | 3 |
|  | 19.44 |  |  |  | [M-H]- | 579.1715 | 271.06088，151.00243，459.11538，119.04882 |  |
| 108 | 21.3 | Liquiritigenin | C_15_H_12_O_4_ | 254.253 | [M+H]+ | 255.0651 | 137.02339，145.02859，68.99727，81.03355 | 2 |
|  | 21.33 |  |  |  | [M-H]- | 253.0501 | 117.03316，91.01741，118.03645，153.01825 |  |
| 109 | 21.56 | Lobetyolin | C_20_H_28_O_8_ | 396.4 | [M-H+FA]- | 441.1766 | 59.01250，71.01242，143.07022，89.03827 | 3 |
| 110 | 22 | Isoliquiritin | C_21_H_22_O_9_ | 418.394 | [M+H]+ | 419.1328 | 137.02324，257.08044，147.04391，119.04918 | 3 |
|  | 22.03 |  |  |  | [M-H]- | 417.119 | 148.01529，255.06590，135.00742，119.04889 |  |
| 111 | 22.33 | Baicalin | C_21_H_18_O_11_ | 446.4 | [M+H]+ | 447.0914 | 271.05908，242.06273，272.06295，241.04922 | 3 |
|  | 22.21 |  |  |  | [M-H]- | 475.1243 | 269.04541，240.03775，136.98682，270.04910 |  |
| 112 | 22.33 | Ononin | C_22_H_22_O_9_ | 430.405 | [M+H]+ | 431.1328 | 269.08038，254.05682，213.09062，237.05421 | 3 |
|  | 22.35 |  |  |  | [M-H]- | 429.1158 | 252.04233，267.06613，223.03922，195.04463 |  |
| 113 | 22.87 | Liquiritigenin | C_15_H_12_O_4_ | 256.25 | [M+H]+ | 257.0803 | 137.02318，81.03340，91.05412，119.04902 | 3 |
|  | 22.92 |  |  |  | [M-H]- | 255.066 | 119.04884，91.01746，93.03308，120.05206 |  |
| 114 | 23.69 | Luteolin | C_15_H_10_O_6_ | 286.236 | [M+H]+ | 287.0549 | 153.01842，135.04413，68.99728，89.03864 | 1 |
|  | 23.71 |  |  |  | [M-H]- | 285.0401 | 133.02814，65.00181，107.01241，151.00229 |  |
| 115 | 23.69 | Quercetin | C_15_H_10_O_7_ | 302.236 | [M+H]+ | 303.0495 | 303.04971，153.01819，137.02354，229.04944 | 2 |
|  | 23.78 |  |  |  | [M-H]- | 301.035 | 151.00243，107.01242，65.00184，121.02803 |  |
| 116 | 23.87 | Calycosin | C_16_H_12_O_5_ | 284.263 | [M+H]+ | 285.0755 | 213.05457，137.02341，134.03624，270.05188 | 2 |
|  | 23.88 |  |  |  | [M-H]- | 283.0609 | 211.03917，268.03723，91.01743，239.03429 |  |
| 117 | 24.12 | 3,8-Di-O-methylellagic acid | C_16_H_10_O_8_ | 330.246 | [M+H]+ | 331.0444 | 316.02118，299.01880，271.02356，228.00522 | 1 |
|  | 24.17 |  |  |  | [M-H]- | 329.0301 | 270.98804，298.98285，242.99294，214.99763 |  |
| 118 | 25.79 | Genistein | C_15_H_10_O_5_ | 270.237 | [M+H]+ | 271.0587 | 225.88614，153.01831，115.05425，91.05427 | 2 |
|  | 25.72 |  |  |  | [M-H]- | 269.0452 | 117.03310，65.00185，107.01237，151.00233 |  |
| 119 | 25.84 | Naringenin | C_15_H_12_O_5_ | 272.25 | [M+H]+ | 273.0751 | 153.01813，147.04395，119.04907，91.05413 | 3 |
|  | 25.85 |  |  |  | [M-H]- | 271.0611 | 119.04886，65.00191，63.02265，107.01247 |  |
| 120 | 25.68 | Apigenin | C_15_H_10_O_5_ | 270.237 | [M+H]+ | 271.0599 | 225.88614，153.01831，115.05425，91.05427 | 2 |
|  | 25.71 |  |  |  | [M-H]- | 269.0452 | 133.02817，63.02260，65.00185，131.04887 |  |
| 121 | 26.13 | Kaempferol | C_15_H_10_O_6_ | 286.236 | [M+H]+ | 287.0546 | 287.05472，153.0183，121.02851，68.99724 | 2 |
|  | 26.15 |  |  |  | [M-H]- | 285.0402 | 93.03314，117.0331，143.04906，65.00178 |  |
| 122 | 27.55 | Isoliquiritigenin | C_15_H_12_O_4_ | 256.25 | [M+H]+ | 257.0802 | 137.02321，81.03342，91.05415，119.04901 | 3 |
|  | 27.57 |  |  |  | [M-H]- | 255.0659 | 119.04889，91.01749，93.03310，120.05206 |  |
| 123 | 27.97 | Formononetin | C_16_H_12_O_4_ | 268.264 | [M+H]+ | 269.0806 | 68.99714，197.05948，118.04115，253.04919 | 3 |
|  | 27.99 |  |  |  | [M-H]- | 267.0661 | 252.04205，223.03937，195.04419，132.02037 |  |
| 124 | 29.6 | Medicarpin | C_16_H_14_O_4_ | 270.28 | [M+H]+ | 271.0587 | 91.05424，153.01822，119.04929，271.05981 | 2 |
|  | 29.54 |  |  |  | [M-H]- | 269.0815 | 137.05969，109.06477， 79.05421，95.04919 |  |
| 125 | 29.85 | Chrysin | C_15_H_10_O_4_ | 254.24 | [M+H]+ | 255.0646 | 153.01811，68.99714，103.05412，129.03333 | 3 |
|  | 29.85 |  |  |  | [M-H]- | 253.0502 | 63.02267，65.00192，143.04897，119.04903 |  |
| 126 | 30.19 | 6-Gingerol | C_17_H_26_O_4_ | 294.4 | [M-H]- | 293.1755 | 283.06097，253.05023 | 3 |
| 127 | 30.27 | oroxylin A | C_16_H_12_O_5_ | 284.26 | [M+H]+ | 285.0751 | 270.05157，186.01561，168.00502，68.99712 | 3 |
|  | 30.26 |  |  |  | [M-H]- | 283.061 | 109.99956，268.03741，165.98959，139.05402 |  |
| 128 | 31.08 | Licoricesaponin G2 | C_42_H_62_O_17_ | 838.9 | [M+H]+ | 839.4047 | 469.33038，487.34076，451.31982，141.01794 | 3 |
|  | 31.19 |  |  |  | [M-H]- | 837.3909 | 351.05673,193.03450 |  |
| 129 | 31.88 | Glycyrrhizic acid | C_42_H_62_O_16_ | 822.404 | [M+H]+ | 823.4094 | 453.33539，435.32422，471.34558，454.33862 | 3 |
|  | 31.89 |  |  |  | [M-H]- | 821.3964 | 351.05673，193.03445，113.02304 |  |
| 130 | 37.06 | Glycyrrhetinic acid | C_30_H_46_O_4_ | 470.684 | [M+H]+ | 471.3459 | 107.08542,149.09598,189.16359,235.16893 | 3 |
|  | 37.09 |  |  |  | [M-H]- | 469.3313 | 425.34161,355.26337 |  |
