# Supplementary material for: Analysis of the chemical constituents and their metabolites in Orthosiphon stamineus Benth. via UHPLC-Q exactive orbitrap-HRMS and AFADESI-MSI techniques
Source: PLoS One. 2024 Jun 25;19(6):e0304852. doi: 10.1371/journal.pone.0304852 (PMC11198764; doi:10.1371/journal.pone.0304852)
Supplement: S3 Table — (DOCX) [file pone.0304852.s007.docx]

**S3 Table Mean peak intensities of compounds identified in blood and kidney tissues in control and administered groups**

| **No.** | **t/min** | **Plausible identity** | **ionic strength(serum CON)** | **ionic strength(serum OS)** | **ionic strength(kidney CON)** | **ionic strength(kidney OS)** |
| --- | --- | --- | --- | --- | --- | --- |
| 1^a,b,c^ | 3.88 | Danshensu | 7.48E+04 | 1.92E+08 | 2.37E+05 | 2.51E+08 |
| 2^a,b,c^ | 4.67 | protocatechuic acid | 1.38E+05 | 1.65E+08 | 1.19E+05 | 3.48E+05 |
| 4^a,b^ | 7.13 | Protocatechualdehyde | 3.03E+05 | 2.31E+09 | / | / |
|  | 7.14 |  | 9.08E+04 | 1.19E+05 | / | / |
| 5^a,b,c^ | 8.96 | Chlorogenic acid | 3.27E+05 | 7.07E+05 | 3.19E+05 | 8.39E+06 |
|  | 9 |  | 0.00E+00 | 1.07E+04 | 7.13E+06 | 3.66E+07 |
| 7^a,b,c^ | 9.82 | Cryptochlorogenic acid | 3.22E+05 | 7.70E+05 | 3.19E+05 | 8.39E+06 |
|  | 9.87 |  | 6.54E+03 | 7.67E+04 | 4.87E+04 | 5.77E+05 |
| 8^a,b,c^ | 9.94 | Caffeic acid | 3.24E+05 | 6.33E+06 | 1.78E+05 | 2.18E+06 |
|  | 9.94 |  | 2.20E+04 | 7.67E+04 | 1.32E+05 | 1.21E+07 |
| 9^a,b,c^ | 13.3 | cis-4-coumaric acid | 4.02E+04 | 4.42E+05 | 1.26E+05 | 1.89E+07 |
|  | 13.34 |  | 1.01E+05 | 4.59E+05 | 3.18E+05 | 1.14E+06 |
| 11^b,c^ | 15.17 | ferulic acid | 4.02E+04 | 5.06E+05 | 1.55E+05 | 6.66E+05 |
|  | 15.18 |  | 2.07E+04 | 4.64E+04 | 1.70E+05 | 2.70E+06 |
| 12^a,b^ | 15.25 | Chicoric acid | 8.04E+03 | 3.05E+04 | / | / |
| 13^a,b^ | 16.03 | Rutin | 4.57E+05 | 1.70E+06 | / | / |
|  | 16.03 |  | 0.00E+00 | 3.05E+04 | / | / |
| 16^a,b^ | 20.1 | Rosmarinic acid | 4.97E+04 | 6.85E+05 | / | / |
|  | 20.13 |  | 3.66E+04 | 6.03E+04 | / | / |
| 17^a,b^ | 20.72 | Lithospermic acid | 5.55E+04 | 2.78E+05 | / | / |
|  | 20.74 |  | 6.56E+03 | 8.39E+03 | / | / |
| 18^a,b^ | 21.99 | salvianolic acid B | 3.65E+04 | 4.73E+05 | / | / |
|  | 22.02 |  | 9.15E+03 | 2.00E+04 | / | / |
| 20^b,c^ | 25.76 | Salvianolic acid C | 5.52E+04 | 6.65E+05 |  |  |
|  | 25.75 |  | 9.12E+03 | 3.56E+04 | 2.46E+05 | 2.41E+06 |
| 21^a,b,c^ | 28.42 | Sinensetin | 6.30E+05 | 3.61E+07 | 5.78E+04 | 2.76E+06 |
| 22^a,b^ | 28.71 | Eupatorin | 3.11E+06 | 3.55E+06 | / | / |
|  | 28.74 |  | 7.64E+03 | 3.19E+04 | / | / |
| 23^a,b,c^ | 1.27 | Stachyose | 1.07E+06 | 1.41E+06 | 5.12E+05 | 4.17E+06 |
| 24^c^ | 1.29 | Raffinose | / | / | 3.42E+05 | 1.77E+06 |
| 25^a,b^ | 1.37 | Stachydrine | 3.19E+06 | 1.34E+08 | / | / |
| 26^a,b,c^ | 1.38 | Adenine | 6.07E+06 | 1.37E+08 | 1.35E+05 | 2.46E+06 |
| 27^b^ | 1.69 | Nicotinamide | 6.25E+05 | 3.44E+07 | / | / |
| 28^a,b,c^ | 1.72 | Adenosine | 5.75E+05 | 1.15E+06 | 5.43E+05 | 2.62E+06 |
| 30^a,b,c^ | 1.77 | L-Tyrosine | 5.90E+05 | 4.58E+08 | 7.37E+05 | 2.80E+07 |
| 31^a,b^ | 1.77 | p-Coumaric acid | 6.07E+05 | 1.12E+08 | / | / |
| 32^a,b,c^ | 1.79 | Guanine | 5.75E+05 | 1.15E+07 | 6.40E+05 | 3.80E+06 |
| 33^a,c^ | 1.78 | Guanosine | / | / | 6.43E+04 | 1.75E+06 |
|  | 1.8 |  | / | / | 1.02E+05 | 1.21E+06 |
| 34^a,b,c^ | 3.29 | L-Phenylalanine | 3.93E+05 | 6.82E+08 | 1.63E+06 | 4.90E+07 |
| 37^a,b^ | 5.88 | Caftaric acid | 2.52E+05 | 2.94E+06 | / | / |
| 38^a,b,c^ | 6.5 | L(-)-Tryptophan | 3.24E+05 | 6.74E+08 | 3.27E+06 | 7.07E+07 |
| 39^b,c^ | 8.85 | 1-Caffeoylquinic acid | 7.66E+04 | 2.96E+06 | / | / |
| 40^b,c^ | 8.85 | scopolin | 3.16E+05 | 8.03E+06 | 2.13E+05 | 5.79E+06 |
| 41^b^ | 9.48 | Eleutheroside B | 3.72E+05 | 1.72E+06 | / | / |
| 44^b,c^ | 11.31 | Gentiopicroside | 3.72E+04 | 6.55E+04 | 6.45E+04 | 5.18E+05 |
| 45^b^ | 12.52 | 4'-O-Glucosylvitexin | 1.62E+04 | 4.35E+05 | / | / |
| 46^a,b^ | 12.52 | Vicenin-2 | 5.36E+04 | 3.69E+05 | / | / |
|  | 12.52 |  | 1.62E+04 | 4.35E+05 | / | / |
| 48^b^ | 13.52 | 3-n-Butylphathlide | 4.02E+05 | 5.40E+06 | / | / |
| 49^b^ | 13.52 | alpha-Asarone | 4.01E+05 | 1.21E+06 | / | / |
| 52^c^ | 16.04 | Isovitexin | / | / |  |  |
|  | 16.04 |  | / | / | 2.51E+05 | 8.02E+05 |
| 62^b^ | 18.51 | Fisetin | 5.27E+05 | 2.88E+06 | / | / |
| 63^c^ | 19.14 | Tracheloside | / | / | 1.13E+05 | 3.69E+06 |
| 68^c^ | 20.12 | Irigenin | / | / | 8.30E+04 | 2.92E+06 |
| 74^a,b^ | 23.48 | Methyl rosmarinate | 2.48E+04 | 3.30E+05 | / | / |
| 75^a,b^ | 26.24 | (+)-Nootkatone | 4.54E+05 | 3.55E+06 | / | / |
| 78^a,b,c^ | 26.62 | Gardenin B | 6.59E+05 | 1.43E+06 | 1.55E+05 | 3.01E+06 |
| 79^b^ | 26.66 | Pedunculoside | 8.53E+04 | 3.55E+06 | / | / |
| 82^a,b,c^ | 29.55 | Eudesmin | 7.42E+05 | 9.61E+06 | 2.66E+05 | 1.83E+06 |
| 83^b^ | 29.69 | Nobiletin | 7.45E+05 | 9.42E+06 | / | / |
| 85^b^ | 30.14 | 6-Shogaol | 7.97E+05 | 1.35E+06 | / | / |
| 86^b,c^ | 30.9 | Senkyunolide A | 7.97E+05 | 9.15E+06 | 7.23E+05 | 9.27E+06 |
| 90^a,b^ | 36.01 | alpha-Linolenic acid | 5.60E+05 | 3.38E+06 | / | / |
| 92^c^ | 36.34 | Ursonic acid | / | / | 4.24E+06 | 6.80E+07 |
