# Supplementary material for: Analysis of the chemical constituents and their metabolites in Orthosiphon stamineus Benth. via UHPLC-Q exactive orbitrap-HRMS and AFADESI-MSI techniques
Source: PLoS One. 2024 Jun 25;19(6):e0304852. doi: 10.1371/journal.pone.0304852 (PMC11198764; doi:10.1371/journal.pone.0304852)
Supplement: Attachment — Submitted filename: Response to Reviewers.docx [file pone.0304852.s009.docx]

Reviewer #1: Dear Authors

The study deals with a meaningful and important question, so the topic interests certain readers in the Plos One. However, the following comments should be addressed because some parts should be revised and improved.

1.In your title, are there any differences between the terms chemical constituent and metabolite?

Yes, chemical composition refers to the prototype of a compound measured in *vitro* such as in aqueous decoctions or powdered preparations, which exists as a prototype in the blood or tissues after administration, and metabolites, which are products resulting from metabolic reactions of the prototypical ingredient in the blood(1, 2). This metabolic operation consists of three distinct phases. Phase I metabolism encompasses functionalization reactions while Phase II involves a series of conjugation reactions. Phase III is denoted by transporter-mediated elimination of drugs and/or metabolites from the body, typically executed by the liver, gut, kidney, or lungs. The metabolites produced by prototypes in the metabolic reactions of phase I can be directly excreted or re-excreted after going through the metabolic reactions of phase II, while also excreted through the metabolic reactions of phase II directly.(3)

2.Did you use a QC sample in your LC-MS/MS analysis? If not, please explain it.

I did not use QC samples, in order to ensure the stability of the instrument and the reproducibility of the data, I prepared and tested the samples separately at different times, and observed their TIC plots, the overall profile and retention time did not find any significant changes, and combined with the comparison of the BLANK samples, the baseline is smooth, so it ensures that there are no residuals and the data can be reproduced. (S1 Fig).

3.In tables 1, 2, and 3 please write the molecular formula correctly like this C9H10O5

Thank you for your suggestion, I have changed the molecular formulae in Tables 1, 2 and 3 and in the appendix to the correct expressions

4. For your annotation of metabolite, add information about error mass to make sure your tentative identification is precise and accurate

Thank you for your suggestion, the Error (ppm) column in the Table 3 is the error value for the molecular mass of the metabolite, all within ±5.

Reviewer #2: This manuscript presents the results of research on the analysis of the chemical compounds content of Orthosiphon stamineus Benth (OS) using UHPLC-Q Exactive Orbitrap- 4 HRMS and AFADESI-MSI techniques. The research is a preliminary stage in utilizing OS as a source of medicine for kidney disease, so that the manuscript compiled is only limited to reporting a list of chemical compounds in OS and metabolites in serum from rats treated with OS extract. In this manuscript there is no in-depth analysis of the data obtained and no detailed discussion of the results of data analysis. Some notes on this manuscript are as follows:

1. The significance of this study only emphasizes the use of UHPLC-Q Exactive Orbitrap-4 HRMS for the analysis of metabolite content of OS and the use of AFADESI-MSI technique to visualize the spatial distribution of compounds in tissues.

Thanks to your suggestion, this paper also compares the compounds contained in the aqueous decoction of OS with those contained in the powder of OS and finds the necessity of decoctions of traditional Chinese medicines to co-analyse the chemical characterisation of OS not only in *vivo* and in *vitro*, but also at the spatial level to characterise the distribution of OS. The UHPLC-Q Exactive Orbitrap-HRMS technique was developed for the first time for the characterisation of kidney tea with good reproducibility and stability, and maximised the extraction of the compounds contained in OS.

2. The method did not describe which part of OS was used and how many biological replicates and technical replicates were conducted in this study.

The dry ground portion of the OS was used and three samples were prepared in parallel for each assay and a total of three replications were performed at different times to clarify the stability as well as the accuracy of the method. (S1 Fig.)

3. The manuscript does not show data on the content of chemical compounds and metabolites in serum and kidney from rats that did not receive OS extract treatment (control group), so it cannot be compared between the content of chemical compounds and metabolites in the two treatments.

The metabolic content (i.e. peak intensity or peak area) has been demonstrated in Table 3 and Appendix F, showing that the control serum and kidney tissue homogenate samples had low levels of the prototypical substance, flush with baseline and using the extracted ion-pairing approach as a complete peak that could appear, but was present in the drug-containing serum and kidney tissue. In contrast, the peak areas of the compound metabolites were all zero in the control group, indicating their specificity.

4. The discussion is only limited to page 25, lines 557 - 580. Even then there are confusing parts (lines 565-571). The author illustrates compound M21 at the beginning of the paragraph, but then suddenly compound M21 is explained with M18 with precursor found at m/z342.09508, which belongs to M21. In explaining the mechanism of change of chemical compounds, the authors did not use literatures as a reference.

Sorry for the confusion due to my mistake, the paragraph has always been about M21, as the numbering was changed during the writing process, here is my mistake. References are cited by accession.

5. Appendix B table starts from No. 93, instead of 1. There seems to be something missing.

There is no loss of data, I am sorry for the misunderstanding because of my numbering, the first 92 numbers are compounds present in the OS, because of the display and labelling of the standard's graphs, i.e. Figures 3 and 4, if the numbering starts from 1 it is easy to confuse, so the compounds present in the OS are numbered together with compounds that are not present.

6. Authors must be able to prove that the chemical analysis in OS was carried out from at least 3 biological replicates and at least 2 technical replicates.

Thank you for the reminder, the duplicate experimental plots have been added to the attachment. (S1-4 Fig)

7. Authors should add more detail discussion regarding the mechanism of metabolite changes in serum and kidney and compare between control and treatment groups.

Thank you for your suggestion, which has been added to the discussion section. Complemented the way of rutin produces metabolites in blood. It has been stated in the literature that the main metabolic pathways involved in rutin in vivo are methylation, glucuronidation, sulphate esterification and their complex reactions(4). M26 and M29 are metabolites produced by different metabolic reactions of rutin. The precursor ion [M+K]+ of M26 occurs at m/z 393.07917，It is possible that a brassinose group (C12H20O9) was first lost, and the reaction was followed by oxidation, methylation and continued loss of CH2O2. The precursor ion [M-H]+ of M29 occurs at m/z 541.02960，The prototypical rutin first binds a glucuronic acid and oxidation reaction at m/z 477 based on the presence of a sulphuric acid and a methyl group at m/z 542 later, in accordance with the literature(4).

**References:**

1.	Liu YN, Hu MT, Qian J, Wang Y, Wang SF. Characterization of the chemical constituents of Jie-Geng-Tang and the metabolites in the serums and lungs of mice after oral administration by LC-Q-TOF-MS. Chin J Nat Med. 2021;19(4):284-94. http://doi.org/10.1016/S1875-5364(21)60028-6

2.	Xu T, Li S, Sun Y, Pi Z, Liu S, Song F, et al. Systematically characterize the absorbed effective substances of Wutou Decoction and their metabolic pathways in rat plasma using UHPLC-Q-TOF-MS combined with a target network pharmacological analysis. J Pharm Biomed Anal. 2017;141:95-107. http://doi.org/10.1016/j.jpba.2017.04.012

3.	Almazroo OA, Miah MK, Venkataramanan R. Drug Metabolism in the Liver. Clin Liver Dis. 2017;21(1):1-20. http://doi.org/10.1016/j.cld.2016.08.001

4.	Goyal J, Verma PK. An Overview of Biosynthetic Pathway and Therapeutic Potential of Rutin. Mini Rev Med Chem. 2023;23(14):1451-60. http://doi.org/10.2174/1389557523666230125104101
